# Collaborative Expression: Transcriptomics of *Conus virgo* Suggests Contribution of Multiple Secretory Glands to Venom Production

**DOI:** 10.1007/s00239-023-10139-8

**Published:** 2023-11-14

**Authors:** Alexander Fedosov, Carmen Federica Tucci, Yuri Kantor, Sarah Farhat, Nicolas Puillandre

**Affiliations:** 1 Department of Zoology, Swedish Museum of Natural History, Box 50007, 10405 Stockholm, Sweden; 2 Institut Systématique Evolution Biodiversité (ISYEB), Muséum National d’Histoire Naturelle, CNRS, Sorbonne Université, EPHE, Université des Antilles, 57 rue Cuvier, CP 51, 75005 Paris, France; 3 Department of Comparative Biomedicine and Food Science, University of Padova, Viale dell’Università, 35020 Legnaro, Italy; 4 A. N. Severtsov Institute of Ecology and Evolution, Russian Academy of Sciences, 33 Leninski Prospect, Moscow, 119071 Russian Federation

**Keywords:** Venom evolution, Venomous animals, Conotoxin, Conidae, Molluscs

## Abstract

Venomous marine gastropods of the family Conidae are among the most diversified predators in marine realm—in large due to their complex venoms. Besides being a valuable source of bioactive neuropeptides *conotoxins*, cone-snails venoms are an excellent model for molecular evolution studies, addressing origin of key innovations. However, these studies are handicapped by scarce current knowledge on the tissues involved in venom production, as it is generally assumed the sole prerogative of the venom gland (VG). The role of other secretory glands that are present in all *Conus* species (salivary gland, SG) or only in some species (accessory salivary gland, ASG) remains poorly understood. Here, for the first time, we carry out a detailed analysis of the VG, SG, and ASG transcriptomes in the vermivorous *Conus virgo*. We detect multiple transcripts clusters in both the SG and ASG, whose annotations imply venom-related functions. Despite the subsets of transcripts highly-expressed in the VG, SG, and ASG being very distinct, SG expresses an L-, and ASG—Cerm08-, and MEFRR- superfamily conotoxins, all previously considered specific for VG. We corroborate our results with the analysis of published SG and VG transcriptomes from unrelated fish-hunting *C. geographus*, and *C. striatus*, possibly fish-hunting *C. rolani*, and worm-hunting *Conus quercinus*. In spite of low expression levels of conotoxins, some other specific clusters of putative venom-related peptides are present and may be highly expressed in the SG of these species. Further functional studies are necessary to determine the role that these peptides play in envenomation. In the meantime, our results show importance of routine multi-tissue sampling both for accurate interpretation of tissue-specific venom composition in cone-snails, and for better understanding origin and evolution of venom peptides genes.

**Supplementary Information:**

The online version contains supplementary material available at 10.1007/s00239-023-10139-8.

## Introduction

Venoms of marine gastropod mollusks of the family Conidae, the cone-snails, are an untapped resource of bioactive compounds, with unique pharmacological properties, and broad diversity of physiological targets (Olivera et al. 2014). Originally, these were the short disulphide-rich peptides *conotoxins* targeted mainly to ion channels in the nerve system or neuro-muscular synapses that were the ultimate focus of cone-snail venom studies (Robinson et al. 2017; Terlau & Olivera 2004). This strong focus was due to the appealing potential of conotoxins for biomedical applications (Safavi-Hemami et al. 2019; Terlau & Olivera 2004), evident from the devastating physiological effects of venom injection in a vertebrate prey, including in human (Kohn 2018; Olivera et al. 2015). Recent integrated studies, however, revealed that the deadly sting is actually only the last episode of the interaction between the cone-snail and its prey, and the earlier phases of interaction may involve specialized bioactive compounds, structurally quite different from conotoxins. For example, the fish-hunter *Conus geographus* produces a specialized fish type insulin that it releases in water as it forages, to excerpt a hypoglycemic condition in fish preventing its escape (Olivera et al. 2015; Safavi-Hemami et al. 2015). Even more exotic is the prey capture by the worm-hunting *Conus imperialis*, specialized to feeding on fireworms (polychaetes of the family Amphinomidae), which attracts its preys by releasing in water an analog of its preys sexual pheromone (Torres et al. 2021). These discoveries suggest that cone-snail envenomation is a more complex process than previously thought, and it requires an equally elaborated morphological basis.

**Fig. 1:**
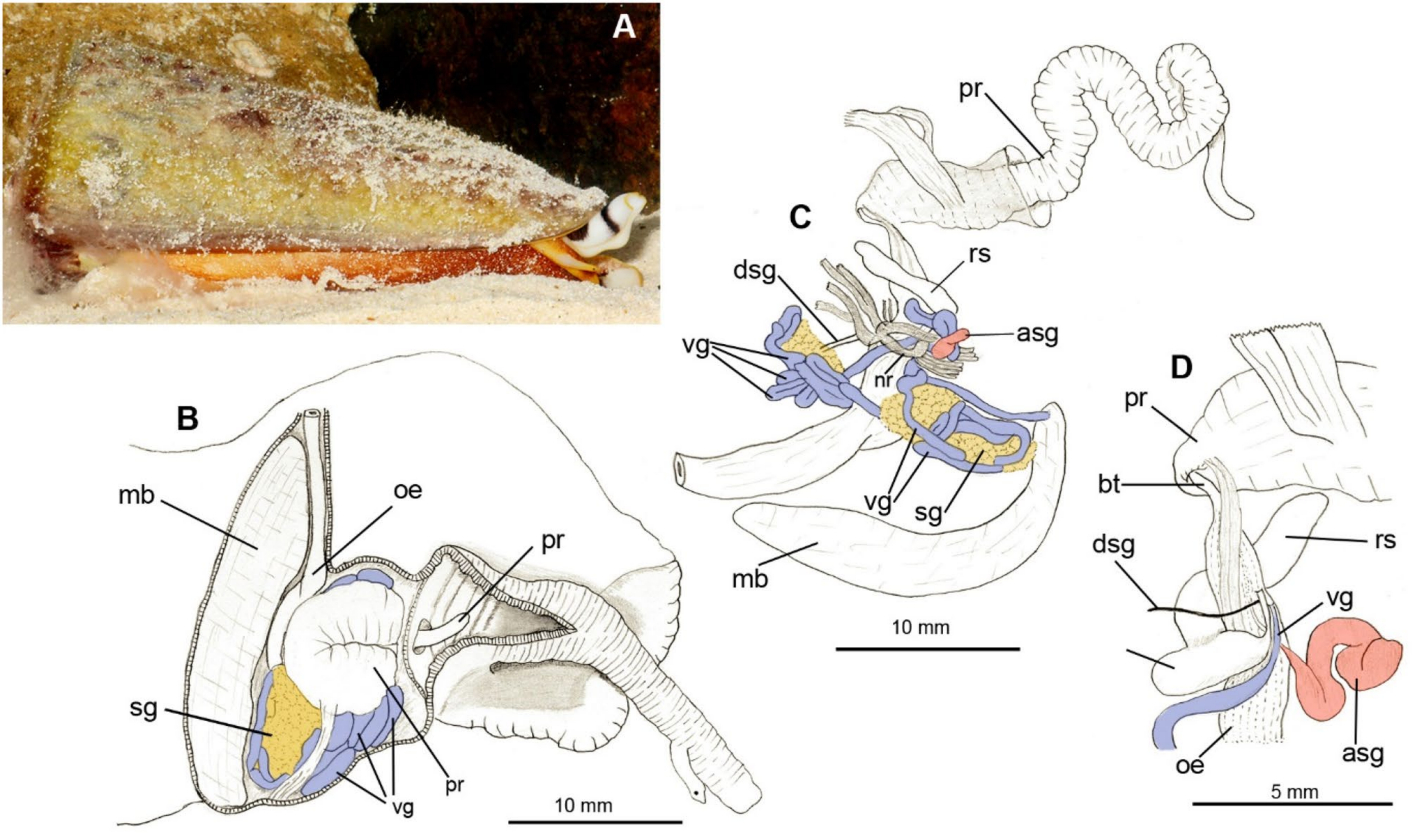
*Conus virgo*. **A** A life specimen of *Conus virgo* Linnaeus, 1758 New Caledonia, Bourail-Poé, 3 m deep. Photo courtesy of David Massemin. **B–D** Foregut anatomy. SG shown in yellow, ASG – in pink, VG in blue. **B** General topology of foregut organs from dorsal side; **C** Foregut complex excised; **D** Close-up of the VG, and ASG at the level of buccal mass. *asg* accessory salivary gland, *bt* buccal tube, *dsg* duct of salivary gland, *mb* muscular bulb, *nr* nerve ring, *oe* esophagus, *pr* proboscis, *rs* radular sac, *sg* salivary gland, *vg* venom gland

Cone-snail envenomation apparatus comprises a tubular venom gland, which produces venom, hypodermic radular harpoons that pierce preys tissues, and an eversible proboscis equipped with a sphincter at its tip to hold and propel a harpoon when executing envenomation (Olivera et al. 2017). This system is functionally wholesome, but the existing knowledge on its functional morphology cannot explain how it can quickly switch between the desired subsets of bioactive compounds, depending on the attack phase, or the type of the external stimuli (Dutertre et al. 2014). One insufficiently explored possibility, the involvement of other foregut secretory glands in the interaction with prey, is the focus of the present study. Cone-snails belong to the superfamily Conoidea within the hyperdiverse molluskan order Neogastropoda (Lemarcis et al. 2022). Neogastropoda, being mainly carnivores, have evolved diverse hunting strategies, often involving specific bioactive compounds (Ponte & Modica 2017; Turner et al. 2018) that can be produced by as many, as four types of foregut secretory glands. These are (i) paired or merged acinous salivary gland(s) (SG), with always paired ducts that open in the pharynx, (ii) paired or single tubular accessory salivary gland(s) (ASG) that open(s) at the tip of the proboscis, (iii) glandular dorsal folds of the mid-esophagus, and (iv) oesophageal gland (= gland of Leiblein) that opens in mid-oesophagus (Kantor 2002; Kuznetsova et al. 2022). The conoidean venom gland derives from the glandular mid-oesophagus merged with the oesophageal gland, and in addition to it, all *Conus* species possess SG, and some predominantly vermivorous species have a large ASG. Whereas both SG and ASG have been shown to produce bioactive compounds in non-conoidean neogastropods (Bigatti et al. 2010; Bose et al. 2017; Gerdol et al. 2018; Kuznetsova et al. 2022; Modica et al. 2015; West et al. 1994), their role in cone snails has been continuously neglected. One exception is the study on *Conus pulicarius* (Biggs et al. 2008), which revealed SG-specific expression of two alpha-conotoxin-like precursors. However, the methodology of this study (cDNA-library analysis) lacked resolution that can now be achieved with RNA-Seq methodology. A recent study (Koch et al. 2022) also demonstrated that SG express venom-related consomatins, but at almost three orders of magnitude lower expression levels, than the VG. Both these studies support the hypothesis that SG secretion may play some role in envenomation. However, there is currently no studies published to comprehensively characterize the set of genes expressed in the SG and ASG based on RNA-Seq data, even though SG transcriptomic datasets have been published for a few *Conus* species recently (Gao et al. 2018, Dutertre et al. 2014; Liao et al. 2022; Koch et al. 2022). To fill this gap, in the present study we generated original RNA-Seq data for VG, SG, and ASG of *Conus virgo* (Fig. 1), and analyzed it alongside with reassembled published RNA-Seq data sets of four further *Conus* species. We show that (i) both the SG and ASG are expressing genes that were previously associated with venomous function, and (ii) their repertoires are distinct from each other, as well, as from the genes expressed in the VG, and vary among species reflecting their phylogeny and/or prey preference. Our results suggest that both, SG and ASG may be involved in the envenomation, and contribute to better understanding of the role of each of them in the prey capture and defense.

## Material and Methods

### Sampling and Dissection

The *Conus virgo* specimen MNHN-IM-2013–54919 (https://science.mnhn.fr/institution/mnhn/collection/im/item/2013-54919) was sampled during the KAVIENG 2014 expedition (expeditions.mnhn.fr), off New Ireland, at depth of 1–3 m. It was photographed and then dissected alive.

*Conus virgo* is characterized by a very elongated head, forming a rostrum with folded walls suggesting its great capacity for extension (Fig. 1). There is a thin funnel-shaped septum separating the posterior part of the proboscis cavity from the body haemocoel (Fig. 1B). The proboscis is very long and coiled, whereas the buccal tube being very narrow as it emerges from posterior proboscis, widens immediately to pass into the rather narrow buccal mass. The venom gland (VG) opens into the buccal mass by a slightly narrowed duct, immediately posterior to the radular sac opening (Fig. 1C, D). The coils of long and rather narrow VG are embedded into an irregularly shaped rather large acinous salivary gland (SG). Paired very thin salivary ducts follow along each other to open at both sides of the proximal radular sac, close to its opening into the buccal cavity. An unpaired, medium-sized, tubular and coiled accessory salivary gland (ASG) is also situated close to the radular sac (Fig. 1D). Its duct is very narrow, following along the buccal mass towards the rear of the proboscis.

During the dissection, the VG was cut at its mid-length, and the proximal and distal parts were immediately suspended in RNA-later solution (Thermo Fisher Scientific, Waltham, MA, USA), and so were the SG and the ASG. All samples were stored overnight at room temperature, and subsequently at − 20 °C.

### RNA Sequencing

RNA was extracted from the four tissue samples separately using the TRIzol reagent (Thermo Fisher Scientific) following the protocol provided by the manufacturer. Bioanalyzer traces were used to assess total RNA quality and determine suitability for sequencing. The cDNA libraries were prepared using the automated mRNAseq stranded library prep protocol at Genome Québec (Montreal, Canada), and paired-end sequenced in the same facility, on a single lane of Illumina HiSeq 2000 with 100-bp long reads.

### Transcripts Assembly and Annotation

Row reads of proximal venom gland (VGP), distal venom gland (VGD), SG and ASG were quality-checked by fastQC v0.11.9 and trimmed using fastp v0.8.3 (Chen et al. 2018) with the following parameters: detect_adapter_for_pe, –trim_front1 10, –trim_front2 10, –trim_tail1 3, –trim_tail2 3, n_base_limit 0, –trim_poly_g, -trim_poly_x, –cut_front, –cut_tail, –length_required 75, –cut_mean_quality 24. The trimmed reads of the four samples were assembled using Trinity v2.13.2 (Grabherr et al. 2011) setting the kmer size to 31 as recommended for the cone-snails venom glands transcriptomes (Li et al. 2017), and using the option –min_contig_length 150 to retain the shorter contigs that may correspond to toxin transcripts. To evaluate assembly quality, its basic metrics were checked using the TrinityStats.pl script, and the trimmed reads were mapped to it with RSEM v.1.3.1 (Li & Dewey 2011). The assembly completeness was evaluated by running BUSCO v.5.2.2 (Manni et al. 2021) against the metazoa_odb10 database. Finally, the transcriptome was filtered to remove mitochondrial and ribosomal sequences whose high expression levels could mask the transcripts of interest.

To identify putative venom-related transcripts we performed a BLASTx run (v.2.8.1, default parameters) on our assembly against an in-house database of animal toxins, with emphasis on those of gastropods (Fedosov et al. 2021). The BLAST hits were first filtered to keep only those with a PID value no less than 60%, the aligned region lacking stop-codons, and no less than 30 amino acids (aa) long. Because the VG transcriptome of *C. virgo* was previously annotated by (Phuong et al. 2016), the set of toxins identified by them was used as a reference to evaluate efficiency of conotoxin detection in our own data. The BLASTx annotation was complemented by a structural similarity search following the methodology of (Fedosov et al. 2021). Possible coding DNA sequences (CDS), corresponding to 35 aa or longer peptides were first predicted by ORFfinder (Wheeler 2003). Those CDSs, containing a signal-region, characteristic of secreted peptides (identified by SignalP-5.9—Nielsen 2017), with D-value >  = 0.7), but lacking transmembrane domains (Phobius v. 1.01—Käll et al. 2007) were annotated against the P-fam HMM database using HMMER (Finn et al. 2011), and by a BLASTp search against the manually curated Uniprot database (The UniProt Consortium 2021). All putative transcripts with available annotations were assigned to five major classes, based on their structural properties and relevance to envenomation:

- Canonical conotoxin superfamilies (denoted by latters A–Y, sometimes with the addition of a digit);
- Minor gene superfamilies—those clusters of conopeptides whose members share structural features of canonical conotoxins, but are discovered recently and are less studied;
- Other conopeptides that do not share key structural features of conotoxins, but are known to contribute to envenomation (e.g. insulin, conkunitzin, contryphan etc.), in particular in cone-snails;
- Other venom components—classes of peptides implicated in the venom function, but not typical of cone-snails venoms;
- enzymes involved in maturation of venom (e.g. disulphide isomerase), or possessing lytic activity, and through that directly or indirectly contributing to envenomation.

The precursor structure was predicted by aligning putative toxins to their closest previously annotated matches accessed from the Conoserver database (Kaas et al. 2012), and supplementary data of (Abalde et al. 2018, 2021; Pardos-Blas et al. 2019, 2022). For selected transcripts, domain structures of the putative precursors were visualized using SMART webtool (http://smart.embl-heidelberg.de/). Prediction of the spatial conformation of mature peptide domains in the interesting venom components was performed using AlphaFold2 (Jumper et al. 2021), available in web browser implementation (https://colab.research.google.com/github/sokrypton/ColabFold/blob/main/AlphaFold2.ipynb). Reliability of the predictions were assessed based on the pLDDT (Local Distance Difference Test) plots, and only those predictions, with average pLDDT values exceeding 60 were considered as reliable. The best scoring model out of five proposed, was refined with Amber, built in the ColabFold pipeline, and visualized using Chimera v.1.15 (Pettersen et al. 2004). The structure similarity search was performed by web-implementation of Foldseek (van Kempen et al. 2023) available at (https://search.foldseek.com/).

### Evaluation of Transcript Tissue Specificity

We run RSEM v.1.3.1 (Li & Dewey 2011) to map the individual samples reads to the single assembly, and to obtain the transcripts-per-kilobase-million (TPM) estimates of expression levels for each of the four *Conus virgo* tissues. The across samples expression data were filtered to remove possible cross-contaminations: if a CDS showed TPM expression level ≤ 1% in one sample relative to some other one (given that all samples were sequenced together), the former expression record was considered erroneous, and replaced by zero. The lack of replicates precluded a statistically robust differential expression analysis, therefore, we focused on the individual transcripts or clusters of transcripts, in which TMP differences among tissues constituted an order of magnitude or more, and with emphasis on the transcripts with high or very high expression in at least one tissue (TPM ≥ 100).

### Transcriptomes of Further *Conus* Species

To corroborate the tissue specific patterns of peptide expression inferred from the vermivorous *Conus virgo*, we accessed VG and SG transcriptomic datasets of five further species: two distantly related fish hunters, *Conus geographus* (Koch et al. 2022) and *C. striatus* (Liao et al. 2022), a presumed fish hunter *C. rolani* (Koch et al. 2022), a mollusk hunter *C. episcopatus* (Lavergne et al. 2015), and one more vermivore, *C. quercinus* (Gao et al. 2018). For each species, the reads of the two tissues were assembled during a single Trinity run, and BUSCO was used to assess the assembly quality. With only 5.8% BUSCO completeness, the *C. episcopatus* assembly appeared to be an outlier, and therefore we do not analyze it further. For the four other datasets, TPM expression levels in each tissue were inferred using the same method as for *C. virgo*. We added the annotated transcripts of *C. virgo* to the in-house toxin database, and used thus expanded database for BLASTx annotation of the four additional *Conus* assemblies. Among the annotated transcripts, only those that (i) contained an N-terminally complete CDS, (ii) with a high-similarity sequence of at least 35 aa, and (iii) a summed TPM expression level in SG and VG of 1 or more were used for comparisons. For each putative transcript, we calculated the proportion TPM_SG_/TPM_VG_, and we arbitrarily treat all putative transcripts with TPM_SG_/TPM_VG_ values exceeding 0.33 as genuine in SG. However, those transcripts unique to SG, but showing low expression should be interpreted with caution, as they could originate from cross-contaminations from other species that might have been processed alongside with the target species.

Finally, as the goal of the present study is to identify SG and ASG secreted peptides of *Conus virgo* relevant to envenomation, we would disregard those secreted SG and ASG peptides that are likely to act on endogenous targets. We attempted identifying these by reassembling foot transcriptome of *Conus ventricosus* (Pardos-Blas et al. 2021), and by performing a BLASTx matching of all *C. virgo* peptides of interest against this foot assembly (E-value e-10). If the alignment length of the BLAST-hit was ≥ half of the query length, the PID value ≥ 30%, and the TPM expression of the respective contig in the *C. ventricosus* foot transcriptome exceeded 10, we hypothesized that at least those *C. virgo* transcripts, that generated this hit are not venom specific. Unless their targets are established (i.e. if these queries represent physiologically characterized conopeptide clusters), we considered these not to be toxins.

## Results

### Datasets, Assembly and Annotation Metrics

The obtained RNA-Seq datasets for salivary gland (SG), accessory salivary gland (ASG), proximal part of the venom gland (VGP) and distal part of venom gland (VGD) comprised from 50,705,126 to 64,529,221 pair-end reads (ASG and SG respectively), (Table 1, Supp. Data 1). A total of 117,475,812 bases were assembled in 185,849 transcripts containing 143,591 genes with a GC percentage of 42.43. The N50 was 1223 and the average contig length 304 (Supp Data 2). The assembly BUSCO score was 61% of complete sequences.

**Table 1:** Analyzed transcriptomic datasets

Species	Tissue	SRA	Code	Platform	PE reads × 10^6^	% BUSCO	% Reads mapped
*C. virgo*	Salivary gland	SRR25338526	SG	HiSeq 2000	50.7	61	85.8
*C. virgo*	Accessory salivary gland	SRR25338527	ASG	HiSeq 2000	64.5		84.1
*C. virgo*	Venom gland distal	SRR25338528	VGD	HiSeq 2000	54.9		78.5
*C. virgo*	Venom gland proximal	SRR25338529	VGP	HiSeq 2000	55.9		76.2
*C. geographus*	Salivary gland	SRR16493590		HiSeq 2500	13.3	41.5	88.35
*C. geographus*	Venom gland	SRR16493595		HiSeq 2500	54.3		71.19
*C. rolani*	Salivary gland	SRR16493589		HiSeq 2500	11.7	35.3	86.32
*C. rolani*	Venom gland	SRR16493597		HiSeq 2500	35.9		83.62
*C. striatus*	Salivary gland	CNS0538564		NovaSeq 6000	34.1	28.9	84.19
*C. striatus*	Venom gland	CNS0538562		NovaSeq 6000	28.9		83.61
*C. quercinus*	Salivary gland	CNR0030095		HiSeq X Ten	28.6	68.3	65.00
*C. quercinus*	Venom gland	CNR0030094		HiSeq X Ten	28.8		64.19
*C. ventricosus*	Foot	SRR13757741		HiSeq 2000	28.9	31.4	83.91

A total of 150 transcripts corresponding to mitochondrial and ribosomal genes were removed from the assembly. The largest number of transcripts with non-zero expression was detected in the SG (150,765), and the smallest—in the VGD (108,420).

### Venom-Related Transcripts of *C. virgo*

We identified 157 putative venom components, of them 69, mainly conotoxins, were identified only by BLASTx, including 21 hits that corresponded to incomplete CDSs (Table S1). A total of 35 putative venom components were identified by both the BLASTx and the CDS annotation using HMMER, and 53 putative venom components were only revealed through the structure-based annotation, including—remarkably, a complete CDS Cvirgo0000024 of a highly expressed O1-superfamily conotoxin. The conotoxin counts per gene superfamily in general are very similar to those published by (Phuong et al. 2016) (Table S2). A total of 88 unique sequences of canonical gene superfamilies were recovered, and the four most diversified gene superfamilies were I2- (18 transcripts vs 14 in Phuong et al. (2016)), O1- (20 vs 26), O2- (10 vs 13), and T- (9 vs 13) (Table S1). The striking difference, however, is the detection of two highly expressed gene superfamilies, B2- and L-, not present in Phuong’s et al. data. A total of 28 identified transcripts represented in our data 17 minor gene superfamilies and other conopeptide classes, most represented by one or two transcripts only (Table S1). Besides the known groups of *Conus* venom peptides, we identified transcripts representing some other peptide cluster implicated in venom functions, of them the most notable being Multi-ShKT (*Stychodactyla helianthus* K-toxin like), Neuropeptide-F, astacin and other metalloproteases, serpin, calreticulin, prolyl-oligopeptidase, and peptidases C, M, and S (Table S1).

### Tissue Specificity of the Venom Transcripts Expression in *Conus virgo*

We considered a venom gene superfamily to be specific to a tissue if the expression of the superfamily’s transcripts (based on their summed TPM values) in this tissue was at least 10 times higher than in other tissues.

**Fig. 2:**
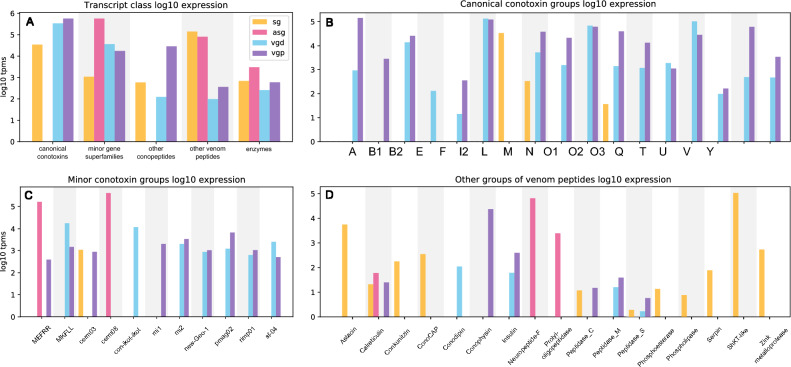
Log10 normalized expression levels of the annotated secretory peptides transcripts in the four analyzed tissues of *Conus virgo*. **A** Major groups of the transcripts; **B** Canonical conotoxin genes superfamilies. **C** Minor conotoxin gene superfamilies; **D** Other groups of conopeptides, and other venom-related peptide clusters

Furthermore, when a gene superfamily is represented by multiple transcripts, we checked how many detected transcripts display the same pattern that we observe at the gene superfamily level. Our results quite expectedly demonstrate that most known conopeptides gene superfamilies show specific expression in VG (Fig. 2A, B), with pronounced partitioning, thereby distal and proximal sections express slightly different repertoires of conotoxin gene superfamilies. However, if Dutertre and coworkers (2014) have taken 12 samples along the venom gland, and so were able to recover a fine-scale picture of VG partitioning, we only had two segments sampled. Nevertheless, 14 conopeptide gene superfamilies in our data show at least tenfold TPM expression difference between VGP and VGD. The transcripts of superfamilies B1-, mi1-, and conophysins are only detected in VGP, whereas those of E-, con-ikot-ikot- and conodipin—only in the VGD. The remaining gene superfamilies are found to be expressed in both, but often with notable differences in the expression levels: e.g. the gene superfamilies A-, V-, to a lesser extent F-, N-, O2-, and O3-. This result is consistent with the existence of two functionally distinct venoms, perhaps a predatory one and a defense-evoked one (Dutertre et al. 2014).

**Fig. 3:**
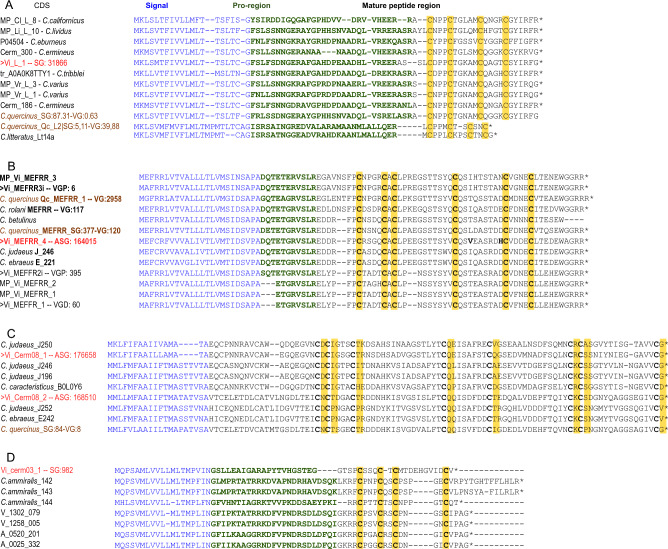
Alignment of the conotoxin transcripts, identified in the SG and ASG of *Conus virgo* (in red), SG and VG of *C. quercinus* (in brown) and in VG of other *Conus species*. **A** L-superfamily; **B** MEFRR-superfamily; **C** Cerm_08 superfamily. **D** Cerm_03 superfamily

Furthermore, some peptide clusters known from *Conus* venom showed specific expression in SG and ASG. First, the L-superfamily conotoxin, Vi_L_1 is detected only in SG, with a very high expression level (TPM_SG_ 34234.51). The CDS shows high sequence similarity (82%) to the L-superfamily conotoxin tr_A0A0K8TTY1 of *C. tribblei* (Barghi et al. 2015), and slightly lower (77–78%) to those of fish-hunting *Conus ermineus* (Abalde et al. 2018) and *Conus magus* M14.9ii (Pardos-Blas et al. 2019) (Figs. 2A, 3A, B). Likewise, the four detected transcripts of the gene superfamily Cerm_08- were expressed exclusively in ASG. Two of them comprised complete CDSs, Cvirgo0000001 and Cvirgo0000005, encoding complete precursors denoted as Vi_cerm08_1—Vi_cerm08_2 respectively, and were among the most highly expressed transcripts overall. Nevertheless, based on the sequence similarity, their closest matches are the venom gland peptides of other *Conus* species: Cvirgo0000001 (Vi_cerm08_1) is 74 PID similar to a Cerm_08- conotoxin of *Conus judaeus* (Pardos-Blas et al. 2022), and Cvirgo0000005 (Vi_cerm08_1) is 67 PID similar to a toxin of *Conus ammiralis* (Abalde et al. 2021) (Figs. 2B, 3C). MEFFR- superfamily is represented in our data by four transcripts, of which Vi-MEFRR_4 has a very high expression in the ASG (TPM_ASG_ 164015), whereas Cv-MEFRR_1, Cv-MEFRR_2i, and Cv-MEFRR_3i—much lower expression in the VG (TPM_VG_ 5 to 396). Whereas the venom gland transcripts were identical or showed highest similarity to the Vi_MEFRR_1 -and Vi_MEFRR_3 identified earlier (Phuong et al. 2016), the ASG transcript is a closer match (87 PID) to the venom gland Cerm_11- superfamily conotoxins of *Conus ebraeus* and *C. judaeus* (Figs. 2B, 3D). One further transcript, Cvirgo0000276 (TPM_SG_ 982) fits into the clusters Cerm_03-6 (gene superfamily Cerm_03) based on the signal sequence and Cysteine framework, it shows a notably longer loop between the 3^rd^ and 4^th^ cysteine residues, compared to the known members of this cluster (Fig. 3D, see also Pardos-Blas et al. 2022). The M- and O2- superfamilies are also detected in SG, however, both represented by the same transcripts as in VG, and at an order of magnitude higher expression levels in the latter. Three transcripts encoding kunitz-domain bearing peptides are identified, of them two (Cv_conkunitzin2 and 3) only detected in VG (TPM_VG_ 70 and 13 respectively), and the third predicted transcript, exclusive in SG (TPM_SG_ 183).

Finally, several transcripts are identified in the *C. virgo* transcriptome that represent some peptide clusters related to venom functions, but are usually not associated with cone-snails venom. These are primarily expressed in SG (Astacin and zink-metalloprotease, CRISP, serpin, but most notably, Multi-ShKT peptide) and ASG (Neuropeptide-Y, prolyl-oligopeptidase).

### Venom-Related Transcripts in the SG Transcriptomes of Other *Conus* Species

The lists of putative venom-related transcripts identified by BLASTx in the assemblies of *Conus geographus*, *C. rolani*, *C. striatus*, and *C. quercinus* are provided in the Tables S3–S6, and the summed TPMs per tissue per transcript cluster—in the table S7. Among them 73, 52, 25 and 57 transcripts, respectively, have higher expression in the SG, compared to the VG. However, only a few of the SG-specific transcripts encode canonical or minor conotoxins (eight, three, two, and 16 in *C. geographus*, *C. rolani*, *C. striatus*, and *C. quercinus* respectively), and only in *C. quercinus*, some SG-specific conotoxin transcripts exceed TPM_SG_ 100.

The conotoxin transcript with the highest genuine expression in SG of *C. geographus* (TPM_SG_ 12.31) belongs to the T-superfamily and shows highest sequence identity to the VG transcript Cerm_322 of *Conus ermineus* (Abalde et al. 2019). The gene superfamilies I1-, I4-, and R- are represented in *C. geographus* transcriptome by one transcript each, either exclusively, or predominantly expressed in the SG, but with low expression levels (Table S3), and therefore unlikely to be functionally significant. The *Conus geographus* SG, however, shows extremely high expression levels of some transcripts classes previously noted in *C. virgo*: Multi-ShKT (one transcript, TPM_SG_ 173495) and Zn-metalloproteinase NAS-15 (one transcript, TPM_SG_ 262839), and lower, yet still rather high expression of porins, kunitz-domain bearing peptides and CRISP-like transcripts (Table S3).

Three conotoxin transcripts with highest expression in SG of *C. rolani* belong to the gene superfamilies B1- (TPM_SG_ 62) and O1- (two transcripts with TPM_SG_ 20 and 11); all three also expressed in VG, with lower or about equal expression levels (Table S4). Similar to *C. geographus*, the gene superfamilies I3- and R- are represented in *C. rolani* by one transcript each, with higher expression in the SG (TPM_SG_ 9 and 2 respectively). Among the non-conotoxin transcripts, four clusters are highly expressed in *C. rolani* SG transcriptome: CRISP-like (one transcript, TPM_SG_ 70955), Zn-metalloproteinase NAS-15 (one transcript, TPM_SG_ 27898), Conohyal-type hyaluronidase (six transcripts with summed TPM_SG_ 57537), and (to a much lesser extent) a kunitz-domain bearing peptide (TPM_SG_ 300).

In *Conus striatus*, Pmag02- transcripts show about equal expression in SG and VG, summing up to TPM ~ 110–115. The R-, O3-, and divergent MKVAVVLLVS-, each is represented by single transcript, with higher expression in SG, but not exceeding TPM_SG_ 25. Among other venom components, most highly expressed are porins, Zink-metalloproteinase NAS15, and CRISP-like transcripts, each showing TPM_SG_ ~ 7000.

In *Conus quercinus*, the gene superfamilies Pmag02- (summed TPM_SG_ 5676), MMLFM- (TPM_SG_ 512), L- (summed TPM_SG_ 237), O2- (summed TPM_SG_ 190) and cerm_08- (summed TPM_SG_ 170) show highest expression in the SG. Of them MMLFM- is specific to SG, while both L-, and cerm_08- while showing higher summed TPM expression levels in SG than in VG, are not specific to the SG. The most highly expressed non-conotoxin transcripts encode astacin (summed TPM_SG_ 2019), Zink-metalloproteinase NAS-15 (summed TPM_SG_ 834), Multi-ShKT(summed TPM_SG_ 554), C-type lectin (summed TPM_SG_ 297), and Kazal-type protease (summed TPM_SG_ 235). It is notable that high relative expression of L-, MEFRR-, Cerm_08- superfamilies conotoxins, astacins, and Multi-ShKT peptides is shared between the *Conus virgo* and *C. quercinus*, however, the direct comparisons of the TPM values are not possible for these two species, as by far more rigorous annotation was performed for the *C. virgo* transcriptome.

### Structure and Putative Functions of Some SG and ASG-Specific Peptides

#### L-Superfamily

The transcript encoding a putative l-superamily conotoxin Vi_L_1 accounts for 95% of the total conotoxin expression inferred from the TPM values, and for 18% of the annotated secreted peptides expression in SG of *Conus virgo*. The predicted 74 aa long precursor comprises a 26 aa mature region with a characteristic for L-superfamily framework 14 (C–C–C–C), and bears a C-terminal RF-amide motif (Fig. 3A, B). There are only three mismatches in the mature region compared to the one in *Conus tribblei* transcript tr_A0A0K8TTY1. No l-superfamily transcripts were detected in the analyzed transcriptomes of either *Conus geographus*, or *C. striatus* and only a divergent, very low expression transcript was detected in VG of *C. rolani*. However, in *C. quercinus*, in addition to the Qc_L2 (Fig. 3A), expressed mainly in the VG, two further L-superfamily transcripts, differing from one another in two aa only, are identified in SG (Fig. 3A). These match the SG transcript of *C. virgo* in length and spacing between Cys residues in the mature region, and show a 71% sequence identity to it. The predicted 3D structure of Vi_L_1 showed notable similarity to that of Fla14.1, an L-superfamily conotoxin of another *Virgiconus* species, *Conus flavidus* (Supp. Data 6) even though the PID between the two was 40.9% only. Although their TPM expression levels are much lower, the overall expression of the L-superfamily in SG of *C. quercinus* is 5 times higher than in VG. Future analysis of SG proteomes will be crucial to validate the L-superfamily conotoxins expression in this tissue. The L-superfamily conotoxins have been found in several species of cone snail, both worm- and fish-hunters (Robinson et al. 2014), and including the small phylogenetically divergent species *Pygmeoconus traillii* (Fedosov et al. 2021), but not in *Conus virgo*, and *a fortiori* not in the salivary gland. The available functional data on the L-superfamily conotoxins (lt14a of *Conus litteratus*, and a synthetic peptide derived from lt14a) suggests inhibition of nAChRs, leading to analgesic effect in mice (Sun et al. 2011). The lt14a, however, is notably shorter than Vi_L_1 (13 aa VS 21 aa—Fig. 3A) and comprises a single Cys-Cys bond, therefore, it is unlikely that the same function is shared by the Vi_L_1.

#### MEFRR-Superfamily

The transcript Vi_MEFRR_4 highly expressed in the *C. virgo* ASG encodes a 87 aa long precursor, with a highly similar signal sequence and an identical pro-region to those in *Conus judaeus* J_246 and *C. ebraeus* E_221 (Pardos-Blas et al. 2022) expressed in VG of respective species. The mature region, despite being also very similar to those in the mentioned transcripts (83%), contains three different residues between the Cys4 and Cys5, and is unique among the similar MEFRR- conotoxins in bearing V in the position 29, and H in the position 35 (Fig. 3B). Furthermore, the mature peptide of Vi_MEFRR_4 matches the lengths of the J_246 and E_221, but differs in this respect from other MEFRR- transcripts of *C. virgo*. Interestingly, among the MEFRR- transcripts of *C. quercinus* one is exclusive to VG (TPM_VG_ 2958), another shows higher expression in SG (TPM_SG_ 377 vs TPM_VG_ 120), and both lack these signature residues. The MEFRR- gene superfamily is mainly known from the worm-hunting species of *Conus*. It is entirely lacking from divergent Conidae genera studied to date (Fedosov et al. 2021), and of *Conus* species with other trophic guilds was previously only reported in *C. ermineus* (as superfamily Cerm11, with low expression (Abalde et al. 2018)). The MEFRR superfamily functions remain unknown, but based on our results, it is one of a few conotoxin clusters that can reach high expression in either SG, ASG, or VG.

#### Cerm08-Superfamily

Two complete Cerm08 superfamily transcripts (entire precursor lengths 104 and 110 aa) match exactly the lengths of the putative closely-related transcripts identified in the *Conus* (*Virroconus*) species (Pardos-Blas et al. 2022) and show the same highly conserved arrangement of 10 Cys residues (Fig. 3C). Fourth similar precursor was identified in SG and VG of *C. quercinus*, with 10 times higher expression in SG (TPM_SG_ 84). The predicted mature peptides do not show detectable resemblance to any physiologically relevant animal peptide. Therefore, additional proteomic data is needed to identify the structure and folding of the mature peptide, whereas activity assays are required for the functional annotation of these transcripts.

#### Kunitz-Domain Bearing Peptides

**Fig. 4:**
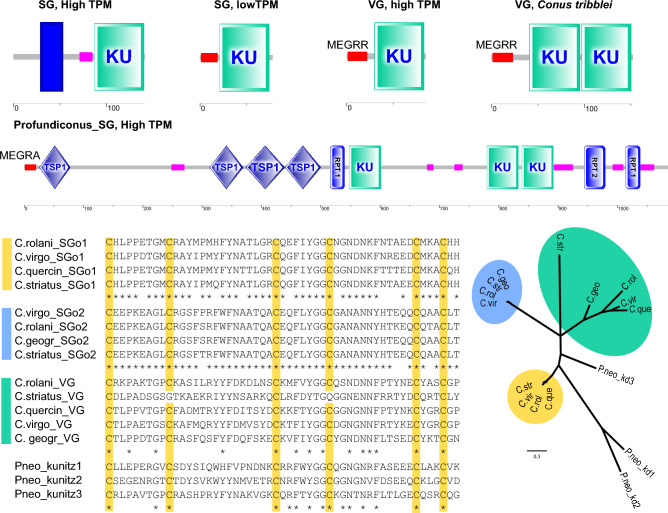
Diversity of the Kunitz-domain encoding transcripts of *Conus* and *Profundiconus*. **A**–**F** Domain structure of domain structure Kunitz-domain encoding transcripts red rectangles—signal sequence, purple rectangles – low complexity regions, blue rectangle—transmembrane domain. **A** *C. virgo* TRINITY_DN2812_c0_g1_i2, SG TPM 183. **B** CDS Cvirgo0008646, SG TPM 13.5. **C** conkunitzin *C. virgo* TRINITY_ DN20510_c0_g1_i1, VG TPM 69.7; **D** *Conus tribblei* conkunitzin tr_A0A0K8TU15; **E** *Profundiconus vaubani* kunitz domain encoding transcript, TSP1—thrombospondin domain, RPT1—internal repeat. **F** Alignment of the kunitz-domains, predicted in the transcripts from three putative orthogroups of *Conus virgo*, *C. geographus* and *C. rolani*, and three consecutive domains detected in the *Profundiconus neocaledonicus* transcript. **G** Maximum likelihood phylogenetic tree of the predicted kunitz-domains

We identified a transmembrane domain preceding the Kunitz-domain in the 141 aa long highly expressed SG transcript, whereas the signal sequence was not detected (Fig. 4A, Supp. Data 3). A 85% identical transcript was detected in SG of *Conus rolani* (TPM 300), and two further very similar transcripts, with 88% and 85% PID—in VG transcriptomes of *C. betulinus* (Peng et al. 2016) and *C. magus* (Pardos-Blas et al. 2019), respectively. None of them possesses a signal sequence typical for the venom con-kunitzins (MEGRR…). A further transcript identified in the SG, but with a notably lower expression (TPM_SG_ 13.5), is shorter (78 aa), lacks the transmembrane domain, and possesses a signal-sequence (Fig. 4B), however, quite different from the one typically reported in the conkunitzins. Three very similar, most probably, homologous transcripts are recovered in the transcriptomes of piscivorous *C. striatus* (TPM_SG_ 2), *C. geographus* (TPM_SG_ 14.8), and possibly piscivorous *C. rolani* (TPM_SG_ 25.7). Finally, the *C. virgo* VG transcript referred to as Vi_conkunitzin_1 by Phuong et al. (2016) is 85 aa long, and contains the signature signal (Fig. 4C), and so do the *true* conkunitzins of *C. rolani*, *C. geographus*, *C. striatus*, and *C. quercinus* (Supp. Data 3). Despite the Kunitz-domain sequences of the SG and VG transcripts of *C. virgo* show only 38 PID, they possess a conserved arrangement of Cysteine residues.

#### Multi-ShKT Transcripts

**Fig. 5:**
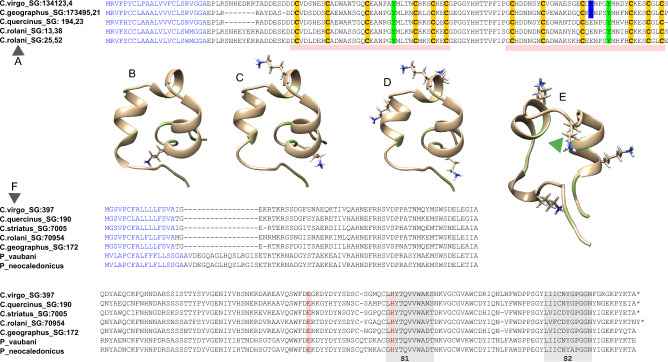
Transcripts of multi-ShKT-like and CRISP-like clusters of *Conus virgo*, *C. geographus* and *C. rolani*. **A** Alignment of the multi-ShKT-like transcripts; ShKT-like domains marked with pink. **B**–**E** Predicted domain conformations of the ShKT-like domains; position of the Cysteine residues indicated in green; orientation of the Lysine residues visualized. **B** *C. virgo*, N-terminal domain, **C** *C. virgo*, C-terminal domain, **D** *C. geographus* C-terminal domain; **E** ShK toxin of *Stychodactyla helianthus*. **F** Alignment of the identified CRISP transcripts; key functional residues (based on Milne et al. 2003), highlighted with pink; conserved signature motifs s1 and s2 marked with grey

The CDS identified by HMMER as a ShKT-like peptide (or multi-ShKT-like referring to Gerdol et al. 2019) is 129 aa long, containing two ShKT-like domains, each spanning 37 aa (Fig. 5A). Each domain comprises six Cys residues, with conserved spacing, and both show disulphide connectivity specific for the ShK toxin: 1–6, 2–4, 3–5, based on the in-silico folding (Fig. 5B–D). Otherwise the sequence similarity between the two domains is rather low—only ~ 43%. Two very similar multi-ShKT-like transcripts are detected in the SG of *C. rolani*, both with rather low expression (TPM_SG_ 12.5 and 25.5), and so unlikely to be functionally important. However, the single multi-ShKT transcript of *Conus geographus* has an extremely high expression in the SG: TPM_SG_ 173495. This transcript features deletion of eight aa between the signal sequence and the first ShKT-like domain (Fig. 5A). Interestingly, the multi-ShKT transcript is also expressed in the SG of *C. quercinus* (TPM_SG_ 194), and it shares the deletion of seven aa residues in the pro-region found in the *C. geographus*. The alignment of the detected multi-ShKT transcripts shows higher sequence variation in the ShKT-like domains and higher conservation in the interleaving regions. In respect to both the precursor structure and the sequences of individual ShKT-like domains, the multiShK-like transcripts in *Conus* closely resemble some of the extremely diversified multi-ShK-like peptides of *Vexillum* (Kuznetsova et al. 2022), with PID up to 62. The proteomic data obtained for *Vexillum* species supported great majority of the sequences proposed based on the transcriptomic data, but those in *Conus* still require validation. It is also unclear, whether they interact with the K + receptors, likewise the *Stychodactyla helianthus* toxin (Fig. 5E). The functional studies (Norton et al. 2004) demonstrated the crucial role of the diad Lysin 22 (marked with green arrow on Fig. 5E) –Arginine 23. Even though all ShKT-like domains of *Conus* are predicted to share the key Arg residue (marked with green on the Fig. 5A), only *C. virgo* and *C. geographus* transcripts encode a Lys residue in a nearby location of the C-terminal domain (Fig. 5C, D). Therefore, further functional studies are necessary to discover molecular targets of these diversified and often highly expressed in the neogastropod SG peptides.

#### CRISP-Like Transcripts

Each of the studied *Conus* species expresses an approximately 190 aa long transcript, annotated as a precursor of Cystein Rich Secretory Peptide (CRISP), with PID ranging from 77 to 82 among the five transcripts. Two similar, most probably, related, peptides were also identified in *Profundiconus neocaledonicus* and *P. vaubani* (Fassio et al. 2019); these, however, differ in the signal sequence, and in possessing an 18 aa-long insertion after it (Fig. 5F). Milne and co-workers (Milne et al. 2003) demonstrated that CRISP Tex31 from venom gland of molluscivorous *Conus textile* has specific proteolytic activity that is consistent with its function being cleaving pro-region from conotoxin precursors. The molecular modeling of the active peptide suggested the key role of Glu115 and His130 in the active center, where His130 is situated within a conserved ‘GHYTQXVW’ motif. In spite of the fact that Tex31 shows low sequence identity with the CRISP of *Conus virgo*, the five identified precursors share the key functional motifs, highlighted by (Milne et al. 2003), and possess the key residues Glu and His in the same alignment positions (Fig. 5F), therefore, we denote them as CRISP-like. All predicted CRISP-like precursors lack a C-rich tail, and so are around 100 aa shorter than Tex31. However, *Conus striatus* is unique among the analyzed species in that according to the available transcriptomic data, it possesses also two slightly divergent transcripts, better matching Tex31 (PID 68.5–69.5) (Supp. data 7). Similar to CRISP-like, these transcripts are SG-specific, but show by far lower expression levels (TPM_SG_ 160) compared to those of CRISP-like (TPM_SG_ 7005). Whereas no proteomic data is currently available to confirm sequences of the CRISP-like transcripts in *Conus* species, their counterparts in *Vexillum* SG (based on the HMMER annotation), were supported by proteomics (Kuznetsova et al. 2022).

## Discussion

### The Venom Gland Does Not Make it all

In the present study we make a first attempt to synthesize data on the transcripts, expressed in the *Conus* foregut glands other than the venom gland. The previous results were generally inconsistent, as to whether conotoxins can be produced anywhere except venom gland. Biggs and coauthors (Biggs et al. 2008) demonstrated tissue-specific expression of some alpha-conotoxins in SG, but their data were only based on the sequencing of cDNA-library clones, a method much less powerful compared to the contemporary RNA-Seq approach. Having analyzed transcriptomes and proteomes of *Conus geographus* SG and radular sac, Dutertre and colleagues (2014) stated that none of these tissues produces conotoxins in the amounts that can be functional. However, the recently published transcriptomic data of *Conus quercinus* (Gao et al. 2018) further supported presence of some conotoxins and other venom-related peptide clusters in the SG, although according to authors, with ‘very small expression levels’.

Our results, establish that both the SG and the ASG express peptide groups associated with venomous functions, and may express canonical conotoxin gene superfamilies, sometimes with very high expression levels. Most notable in *Conus virgo* are the SG-specific L-superfamily transcript (TPM_SG_ 34234.51) and an ASG-specific MEFRR- transcript (TPM_ASG_ 164015). Both L- and MEFRR- superfamilies transcripts are also present, and show moderately high expression in SG of *C. quercinus*, which is phylogenetically close to *C. virgo* (Puillandre et al. 2014). Conversely, neither L- nor MEFRR- transcripts were identified in the SG of the *Conus* species with well-known (*C. geographus*, *C. striatus*) or hypothesized (*C. rolani*) specialization to piscivory. Altogether, these species show low expression levels of canonical conotoxin gene superfamilies in the SG confirming results of Dutertre et al. (2014), and all three of them, to our knowledge, lack ASG.

Finally, perplexing results were published by Pardos-Blas et al. (2021), reporting high expression of some well-known conotoxins (A- and Q-superfamilies), and hormone-like peptides (prohormone-4b, insulin-related peptide 2) in foot of *Conus ventricosus*, prompting a call for re-evaluation of these clusters functions. We used BLASTx against the *C. ventricosus* foot transcriptome with a rather relaxed identity threshold (PID > 30, E-value > E-10), to check whether any of the *C. virgo* SG and ASG peptides of interest have highly expressed counterparts in foot of *C. venticosus*. Only one predicted transcript, encoding an O2- superfamily conotoxin (TPM_VGP_ 9.52), revealed a highly identical counterpart (PID 86), in *C. ventricosus* (TPM_FOOT_ 10.94). Given that *C. ventricosus* is phylogenetically closer to *C. virgo* than any non-vermivore species analyzed here, this results tend to support tissue specificity of the SG and ASG clusters addressed here.

Annotation of SG and ASG-specific transcripts and comparison with their structural counterparts expressed in the VG reveals quite distinct complements of highly expressed peptides. Some peptide classes, such as multi-ShK, astacin, Zink-metalloproteinase NAS-15, CRISP, membrane associated kunitz-domain bearing peptides are specific to the SG. They are always present in a SG transcriptome, at least at ‘baseline’ expression level (100 < TPM < 1000), but may reach higher expression: multi-ShK-like in *C. geographus* and *C. virgo*, Zink-metalloproteinase in *C. geographus*, or CRISP in *C. rolani*. Remarkably, the same peptide clusters are highly expressed in the SG of non-conoidean neogastropods: mollusk-hunting *Vexillum* (Costellariidae) (Kuznetsova et al. 2022) and fish blood-sucker *Colubraria* (Colubrariidae: Buccinoidea) (Gerdol et al. 2019; Modica et al. 2015). Because, with only a few exceptions, SG is homologous, and histologically identical across the Neogastropoda (Ponte & Modica 2017), we can hypothesize that this complement of SG-specific transcripts is shared in all or most neogastropods, probably reflecting original function of this tissue. Some species-specific SG transcripts may add up to this conserved repertoire and reach high expression: L-superfamily conotoxins in *C. virgo* and *C. quercinus*, SG porins in *C. geographus* and *C. striatus,* or Conohyal-type hyaluronidase in *C. rolani*. Therefore, we can suggest that likewise in VG, the SG peptide repertoire varies from species to species to some extent, reflecting phylogeny, or role of the SG in feeding/hunting, or both. The next step in corroborating, whether the peptide clusters identified herein play a role in feeding is the confirmation or negation of their presence in the peptide fractions of both SG and injected venoms.

Because ASG transcriptome data were available only for *Conus virgo*, much less can be deduced about its profile in general. Our results show rather few peptides clusters identified, but with high expression levels, thus suggesting that ASG is highly specialized. ASG transcripts encoding putative secreted peptides sum up to 660,927 TPM expression (compared to 639,332 in VGP, 381,083 in VGD, and only 203,738 in SG), indicating that the major role of the ASG is likely to be similar to that in VG—secretion of rather short peptides (Table S1).

### On the Possible Functions of SG and ASG

Because both SG and ASG open in the anterior alimentary channel, both these structures can utilize the same delivery system as VG, and therefore, participate in the envenomation by injecting venom through the hypodermic radular harpoon. Alternatively, they can be involved in the interaction with the prey or predator more broadly (i.e. by releasing their secretion in water), or carry out other functions related to feeding. To propose sensible hypotheses on the VG, SG, and ASG functions, it is worthwhile to briefly review their structure. Venom gland (VG) is a highly convoluted tube, with rather broad lumen, tall secretory epithelium, and scarce muscular fibers in the external collagenous layer; the discharge of its secretion is achieved through the contraction of the distal muscular bulb. The microstructure of the VG epithelium changes notably from distal to proximal region: whereas the distal part of the gland contains well-defined apical epithelium, bearing dense microvilli, the proximal part, bearing much more numerous venom granules, has poorly defined epithelium, presumably resulting from the intensive holocrine secretion (Dutertre et al. 2014; Marshall et al. 2002). This change of epithelium structure is consistent with different sets of genes expressed in VGP and VGD (Dutertre et al. 2014, current results). In general, with such structure a maximal number of secretory epithelium cells have a contact with the VG lumen, allowing a quick discharge of accumulated venom granules in the lumen, whereas the contraction of the muscular bulb propels it further through the proboscis into the body of a prey or a predator. Accessory salivary gland (ASG) is a paired or unpaired gland, tubular likewise the VG, comprising two layers of epithelium separated by a muscular layer, and its duct lined with non-ciliated epithelium, opens in the anteriormost part of the buccal tube (Kantor 2002). Therefore, in functional aspect the ASG is quite similar to VG: while the lumen is accessible to all or majority of the internal epithelial cells, permitting a quick release of secretory granules, a contraction of the muscular layer would deliver the secretion to the tip of the proboscis, where it is ready to be injected or sprayed in water. The structure of the SG is, however, quite different: the secretory tissue is acinous, with terminal tubules having a narrow, slit-like lumen; muscular fibers are absent, and the very thin salivary ducts are lined with ciliated epithelium. This morphology suggests that the gland is emptied through the secretion flow organized by the coordinated movement of cilia, which one would expect to be much slower than the discharge prompted by the muscular contraction.

The hypothesized functional differences of VG, ASG, and SG do make sense in the light of the broad functional annotation of the most abundantly expressed gene products. The transcriptomes of the quickly emptied VG and ASG are dominated by neuropeptides (conotoxins, other conopeptides targeted to neuronal circuits, including neuropeptide F), whereas SG mostly produces peptides with proteolytic activity. It is therefore likely that VG and ASG are primarily involved in the envenomation (Bigatti et al. 2010; West et al. 1994), whereas the main function of SG may be different, and the most obvious one is the pre-digestion of the prey once it is engulfed in the rostrum. It, however, does not explain the presence of putative neuropeptides L-superfamily conotoxins and multi-ShKT-like precursors in the SG. It is possible that some of the secreted SG peptides may be involved in hunting: they may be transported from the SG to the buccal mass before the active phase of the hunting, similar to a hungry mammal dripping saliva. These peptides are ‘flushed’ by the VG venom stream through the buccal mass, and the mixture of the SG and VG peptides is injected as the muscular bulb contracts. Finally, SG secretion may play a role in maturation of the radular harpoons. A recent ultrastructural study of the *Conus* radular sac (Vortsepneva et al. 2019) demonstrated that radular harpoons once they are formed are not yet hollow – their cavity is filled with cell debris. The SG secretion may be transferred to the radular sac, where its proteolytic compounds may be involved in ‘final cleaning’ of the radular harpoons, emptying their cavity for unobstructed passage of venom. On the contrary, ASG possesses its own muscular component, and opens in the anterior part of the buccal tube, close to the mouth, suggesting that its secretion can be injected or released independently from that of VG. It is therefore possible that the peptides produced by ASG are used either in a different phase of the hunting, or in response to other type of stimuli, as a separate tool of the cone-snail biochemical arsenal. Further studies on the targets of SG and ASG secreted peptides and their transport during hunting and defense are necessary to verify the proposed hypotheses.

### Three Secreting Tissues at Once: Evolutionary Implications and Methodological Warnings

An important aspect of the presence of three tissues potentially contributing to envenomation is that their roles may vary from species to species and evolve to better match a novel prey taxon and/or feeding behavior. In general, novel toxin genes typically derive from non-venom-related ones through gene duplication, followed by the acquisition of novel function, and specific expression in a tissue relevant to envenomation (Hargreaves et al. 2014; Zancolli & Casewell 2020). When as many as three tissues functionally complementary, or partly redundant, are available, one can expect that an increased potential for cross-tissue gene recruitment exists, enhancing development of novel molecular functions and/or redistribution of the existing ones among the tissues. Comparative transcriptomics provide a powerful tool for inferring cross-tissue recruitments and detecting onset of novel functions (Kuznetsova et al. 2022; Safavi-Hemami et al. 2016). As an example here, we consider the diversity of the kunitz-domain encoding transcripts (KDET) in cone-snails.

Conkunitzins are one of the well-established venom components of *Conus*, detected in most studied species, and functionally important as K + channel blockers (e.g. in fish hunting *Pionoconus*—Olivera et al. 2014). The picture obtained for the species studied herein suggests the existence of at least three major orthogroups of KDETs. One of them, expressed mainly in SG, lacks a signal sequence, its peptide products are membrane associated, and there is always only one Kunitz-domain (Fig. 4A). Conversely, KDETs of the second major group lack a transmembrane domain, but possess a signal sequence of a secretory peptide. Similar to the first orthogroup, they are highly conserved, encode single kunitz domain (Fig. 4B), and are expressed in SG. The KDETs of the third orthogroup, are the *true* conkunitzins: they are more variable in both the domain structure (Fig. 4C, D) and the aa sequence of kunitz-domains, and they are expressed in the VG of many cone-snail species, including the most early-diverging genus *Profundiconus* (Fassio et al. 2019). Despite sharing a signal sequence similar to ‘MEGRR- ‘, the *Profundiconus* KDETs are much longer and comprise multiple kunitz-domains, interleaved with long repeats or low complexity regions (Fig. 4E).

Therefore, the high domain conservation in the membrane-associated kunitz-domain bearing peptides and the secreted KDETs of the SG contrasts with the rather variable sequences of the ‘venom’ conkunitzins (Fig. 4F, G), pointing at a different function. This pattern is overall very similar to the prey-preference driven evolution of venom insulins in cone snails (Safavi-Hemami et al. 2016), which were demonstrated to differ drastically from the highly conserved endogenous insulins expressed in the nerve ring. However, future studies are needed to detect molecular targets of the SG-expressed kunitz-domain bearing peptides, and identify their physiological functions. It should be noted that the power of the comparative approach greatly depends on the sampling, and the data availability for SG and ASG is currently the main limiting factor.

It is not only inference of cross-tissue gene recruitment that relies on the accurate information as to which gene superfamilies and orthogroups are present in which species; this data constitutes our knowledge on venom composition, which is commonly perceived to be species-specific. As mentioned above, the transcriptomic data generated from single tissue may be insufficient for either evolutionary inference, or venom composition analysis. For example, despite the gene superfamilies L-, Cerm_08- and MEFRR- are highly expressed in SG / ASG of *C. virgo*, they have been missed or underrepresented in the venom composition inference solely based on the VG profiling (Phuong et al. 2016). Therefore, most *Conus* venom composition analyses published to date, even if based on updated reference databases and rigorous annotation, are likely to be showing only part of the picture, as long as they solely rely on the VG transcriptome. The lack of expression of some peptide cluster in VG may not reflect the ability of a species to produce it in general, and so, presence-absence matrices for venom gene superfamilies, derived from transcriptomics, such as those published by Phuong et al. (2016) or Fedosov et al. (2021) should be taken with some caution.

Furthermore, transcriptomic data is instrumental for matching molecular weights output from mass-spec runs: a sequence lacking in the search database would be basically invisible in the shotgun proteomics data. The missing fraction would expectedly be small, when matching a transcriptome and a proteome of the same tissue; however, otherwise, it may be more significant. In the context of cone-snails venomics studies, this would concern the detection of toxin peptides in the injected venom, if the search databases derive from a VG transcriptome only (Dutertre et al. 2013; Himaya et al. 2018; Himaya et al. 2015). From one hand it allows detecting an injected fraction of the VG-secreted conotoxins – the target group of many studies on cone-snails venom. On the other hand, it may crop too wide margins off the picture, thus simplifying captured venom composition, and ultimately, impeding understanding of the venoms complexity and functions. Indeed, how do we know that the injected venom is secreted solely by VG, and does not comprise a mixture of peptides produced in different foregut glands? One way of addressing this question is to start routinely sequencing other secretory glands of the *Conus* foregut, which is quite achievable, taking into account that (i) it does not require any additional sampling-related efforts and formalities, (ii) costs of High-Throughput Sequencing are currently hardly a limiting factor, and (iii) assembly and annotation protocols for multi-tissue transcriptomes are generally well-established and can to a large extent be automated.

## Conclusion

Our transcriptomic analysis on three secretory foregut glands in *C. virgo* demonstrated that all three of them—venom gland (VG), salivary glands (SG), and accessory salivary glands (ASG)—are expressing toxin genes, and their repertoires are gland-specific. The overlap among the glands is rather limited, at both the individual toxin and gene superfamily level. However, both SG, and ASG show high expression of some transcript clusters, previously thought to be specific for VG, including the conotoxin gene superfamilies L-, MEFRR-, and cerm_08. This supports a hypothesis that both the SG and ASG may contribute to envenomation of a prey by a predator, even though some data suggest that envenomation is not the main function of the SG secretion. Although sequences of the SG and ASG-expressed secreted peptides remain to be verified by proteomic analyses, and their molecular targets are not known, the discovery of the variety of potentially bioactive peptides in these previously neglected glands demonstrates that our knowledge of cone-snail biochemistry is far from being comprehensive. Furthermore, it has important implications for methodology and data interpretation in future studies on cone-snails venom—especially those aligning by default the composition of the injected venom with the transcriptomic library derived from VG only. We hope that our findings will make a convincing argument to start routinely profiling broader spectrum of secretory tissues in cone-snails to shed light on the entire complexity of their biochemical arsenal.

### Supplementary Information

Below is the link to the electronic supplementary material.

Supplementary file1 (XLSX 1621 kb)

## Data Availability

All the original Illumina raw of reads files were submitted to NCBI archive under the Bioproject PRJNA992222; the original scripts, and supplementary data are available at https://github.com/SashaFedosov/Conus_virgo.
